# Supplementary material for: Homologous but not heterologous COVID-19 vaccine booster elicits IgG4+ B-cells and enhanced Omicron subvariant binding
Source: NPJ Vaccines. 2024 Jul 17;9:129. doi: 10.1038/s41541-024-00919-8 (PMC11252355; doi:10.1038/s41541-024-00919-8)
Supplement: Supplementary file 1 — Supplemental Information [file 41541_2024_919_MOESM1_ESM.pdf]

Supplementary Tables (n= 7) **and Figures** (n= 9)

Supplementary Table 1. Participants who received a homologous vaccination schedule

**Participant**	**Age (yrs)**	**Sex**	**Primary vaccine**	**Third dose vaccine**	**Timing of confirmed SARS-CoV-2 infection**
1	24	F	BNT162b2	BNT162b2	81 days before 6-months post-dose 3 sample
2	25	F	BNT162b2	BNT162b2	30 days before 6-months post-dose 3 sample
3	25	M	BNT162b2	BNT162b2	NA
4	26	M	BNT162b2	BNT162b2	NA
5	28	F	BNT162b2	BNT162b2	29 days before 6-months post-dose 3 sample
6	31	M	BNT162b2	BNT162b2	NA
7	32	F	BNT162b2	BNT162b2	66 days before 6-months post-dose 3 sample
8	35	F	BNT162b2	BNT162b2	NA
9	37	F	BNT162b2	BNT162b2	Between 1- and 6-months post-dose 3
10	38	F	BNT162b2	BNT162b2	NA
11	39	M	BNT162b2	BNT162b2	20 days before 6-months post-dose 3 sample
12	40	M	BNT162b2	BNT162b2	NA
13	42	F	BNT162b2	BNT162b2	15 days before 6-months post-dose 3 sample
14	45	F	BNT162b2	BNT162b2	NA
15	46	M	BNT162b2	BNT162b2	NA
16	48	M	BNT162b2	BNT162b2	119 days before 6-months post-dose 3 sample
17	51	F	BNT162b2	BNT162b2	119 days before 6-months post-dose 3 sample
18	62	M	BNT162b2	BNT162b2	NA
	37 (24-62) median w range	56% Female		100% BNT162b2	

Supplementary Table 2. Participants who received a heterologous vaccination schedule

Participant	Age	Sex	Primary vaccination	Third dose vaccination	Time of confirmed SARS-CoV-2 infection
19	26	M	ChAdOx1	BNT162b2	26 days before 6-months post-dose 3 sample
20	27	F	ChAdOx1	BNT162b2	12 days before 1-month post-dose 3 sample
21	29	F	ChAdOx1	BNT162b2	38 days before 6-months post-dose 3 sample
22	32	F	ChAdOx1	BNT162b2	NA
23	33	F	ChAdOx1	BNT162b2	NA
24	33	M	ChAdOx1	BNT162b2	Before 6-months post-dose 3 sample
25	34	F	ChAdOx1	BNT162b2	67 days before 6-months post-dose 3 sample
26	35	M	ChAdOx1	BNT162b2	NA
27	36	F	ChAdOx1	BNT162b2	42 days before 6-months post-dose 3 sample
28	37	F	ChAdOx1	BNT162b2	NA
29	37	F	ChAdOx1	BNT162b2	20 days before 6-months post-dose 3 sample
30	38	F	ChAdOx1	BNT162b2	NA
31	42	M	ChAdOx1	BNT162b2	61 days before 6-months post-dose 3 sample
32	43	F	ChAdOx1	BNT162b2	NA
33	45	F	ChAdOx1	BNT162b2	NA
34	47	F	ChAdOx1	BNT162b2	10 days before 6-months post-dose 3 sample
35	48	F	ChAdOx1	BNT162b2	NA
36	48	F	ChAdOx1	BNT162b2	NA
37	48	F	ChAdOx1	mRNA1273	NA
38	50	F	ChAdOx1	BNT162b2	Infection confirmed, no record of date (between 1- and 6-months post dose 3)
39	54	F	ChAdOx1	BNT162b2	NA
40	56	F	ChAdOx1	BNT162b2	NA
41	57	M	ChAdOx1	BNT162b2	NA
42	58	F	ChAdOx1	BNT162b2	15 days before 1-month post-dose 3 sample
43	64	F	ChAdOx1	BNT162b2	NA
	42 (26-64) median w range	80% Female		96% BNT162b2	

Supplementary Table 3. Participant characteristics

	**Homologous**	**Heterologous**	***p*-value**[1]
Timing of blood sampling (days post-vaccination; median w range)			
3-4 weeks post-dose 1	23 (19-31)	28 (25-29)	**0.0012**
1-month post-dose 2	28 (25-35)	28 (27-36)	0.36
6-months post-dose 2	185 (181-194)	178 (154-197)	**0.0003**
1-month post-dose 3	31 (27-43)	29 (28-64)	0.69
6-months post-dose 3	184 (167-212)	184 (129-208)	0.92
Age (years; median w range)	37 (24-62)	42 (26-64)	0.15
% Female	55.6% (10/18)	80% (20/25)	0.09[2]
BNT162b2 at dose 3	100% (18/18)	96% (24/25)	0.39[2]

[1] Non-parametric Mann-Whitney test with Bonferroni correction for multiple comparisons
[2] Chi-square test

Supplementary Table 4. Composition of the antibody panels

	Fluorochrome															
Tube	**BUV395**	**BUV496**	**BUV737**	**BV421**	**BV480**	**BV650**	**BV711**	**BV786**	**FITC**	**PerCP-Cy5.5/ BB700**	**PE**	**PE-Vio615**	**PC7/ PE-Cy7**	**APC**	**AF700**	**APC-H7**
1. TruCount	-	-	-	-	-	-	-	-	CD3	CD45	CD16 + CD56	-	CD4	CD19	-	CD8A
2. Ag-specific Bmem	RBD ancestral	CD3	RBD ancestral	CD27	RBD BA.2	RBD BA.5	CD21	CD71	IgG2 + IgG3	IgD	IgG1 + IgG2	IgA	CD19	IgG4	Viability	CD38
3. Streptavidin control	Strep	-	Strep	CD27	Strep	Strep	-	-	CD3	IgD	-	-	CD19	-	Viability	-

Supplementary Table 5. Antibody list

Marker	Fluorochrome	Clone	Supplier	Cat. number	Volume/ test (µl)	Tube(s)
CD3	BUV496	UCHT1	BD Bioscience	612940	1	2
CD3	FITC	SK7	BD Biosciences	555332	46 ng*	1
CD3	FITC	UCHT1	BD Biosciences	662995	1	3
CD4	PE-Cy7	SK3	BD Biosciences	555332	30 ng*	1
CD8A	APC-Cy7	SK1	BD Biosciences	555332	126 ng*	1
CD16	PE	B73.1	BD Biosciences	555332	33 ng*	1
CD19	APC	SJ25C1	BD Biosciences	555332	46 ng*	1
CD19	PE-Cy7	SJ25C1	BD Biosciences	557835	5	2
CD21	BV711	B-ly4	BD Biosciences	563163	5	2
CD27	BV421	M-T271	BD Biosciences	562513	1	2 / 3
CD38	APC-H7	HB7	BD Biosciences	303534	1	2
CD45	PerCP-Cy5.5	2D1	BD Biosciences	555332	120 ng*	1
CD56	PE	NCAM16.2	BD Biosciences	555332	22 ng*	1
CD71	BV786	M-A712	BD Biosciences	563768	1	2
IgA	PE-Vio615	REA1014	Miltenyi Biotec	130-116-882	1.5	2
IgD	BB700	IA6-2	BD Biosciences	566538	1	2
IgG1	PE	G17-1	BD Biosciences	624049	0.1	2
IgG2	PE	HP6002	BD Biosciences	624049	0.5	2
IgG2	FITC	HP6002	BD Biosciences	624045	1	2
IgG3	FITC	HP6047	BD Biosciences	624045	0.5	2
IgG4	APC	SAG4	Cytognos	CYT-IGG4AP	2	2
Strep	BUV395	-	BD Biosciences	564176	0.67	3
Strep	BUV737	-	BD Biosciences	564293	0.67	3
Strep	BV480	-	BD Biosciences	564876	0.67	3
Strep	BV650	-	Biolegend	405232	0.13	3
Viability	AF700	-	BD Biosciences	564997	0.1	2 / 3

* total amount of antibody in ng per test

Supplementary Table 6. Flow cytometer set-up

LSRFortessa X-20		LSRII		FACSLyric		Fluorochromes used in this study
**355 nm**		**-**		**-**		
379/28	No LP	-	-	-	-	BUV395
525/50	505 LP	-	-	-	-	BUV496
740/35	690 LP	-	-	-	-	BUV737
**405 nm**		**405 nm**		**405 nm**		
450/50	No LP	450/50	No LP	448/45	448/45	BV421
525/50	505 LP	525/50	505 LP	528/45	500 LP	BV480
-	-	586/15	570 LP	-	-	-
610/20	600 LP	610/20	600 LP	606/36	606/36	-
670/30	635 LP	660/20	630 LP	-	-	BV650
710/50	685 LP	710/50	685 LP	715/50	715/50	BV711
780/60	750 LP	780/60	750 LP	755 LP	755 LP	BV786
**488 nm**		**488 nm**		**488 nm**		
488/10	No LP	488/10	No LP	488/15	No LP	SSC
530/30	505 LP	530/30	505 LP	527/32	507 LP	FITC
-	-	-	-	586/42	560 LP	PE
710/50	685 LP	710/50	630 LP	700/54	665 LP	PerCP-Cy5.5, BB700
-	-	-	-	783/56	752 LP	PE-Cy7
**561 nm**		**561 nm**		**-**		
586/15	No LP	582/15	No LP	-	-	PE
610/20	600 LP	610/20	600 LP	-	-	PE-Vio615
675/50	635 LP	685/35	635 LP	-	-	-
780/60	750 LP	780/60	750 LP	-	-	PE-Cy7, PC7
**640 nm**		**640 nm**		**640 nm**		
670/30	No LP	670/14	No LP	660/10	660/10	APC
730/45	690 LP	730/45	690 LP	720/30	705 LP	Fixable Viability Stain 700
780/60	750 LP	780/60	750 LP	783/56	752 LP	APC-H7, APC-Cy7

Supplementary Table 7. Target values for 7th peak of rainbow beads in fluorescent channels

Fluorochrome	Channel	Lower (-15%)	Target MFI	Upper (+15%)	Recommendation
**BUV395**	**UV395**	17,000	**20,000**	23,000	In-house
**BUV496**	**UV525**	23,800	**28,000**	32,200	In-house
**BUV737**	**UV737**	21,2500	**25,000**	28,750	In-house
**BV421**	**V450**	100,452	**118,178**	135,905	EuroFlow
**BV480**	**V525**	93,871	**110,436**	127,002	EuroFlow
**BV650**	**V670**	34,383	**40,450**	46,518	In-house
**BV711**	**V710**	15,079	**17,740**	20,401	In-house
**BV786**	**V780**	1,903	**2,239**	2,575	In-house
**FITC**	**B530**	28,752	**33,826**	38,900	EuroFlow
**PerCP-Cy5.5/ BB700**	**B710**	66,846	**78,642**	90,438	EuroFlow
**PE**	**YG586**	32,381	**38,095**	43,809	EuroFlow
**PE-Vio615**	**YG610**	178,500	**210,000**	241,500	In-house
**PE-Cy7**	**YG780**	8,316	**9,783**	11,250	EuroFlow
**APC**	**R670**	158,639	**186,634**	214,629	EuroFlow
**AF700**	**R730**	121,550	**143,000**	164,450	In-house
**APC-H7**	**R780**	64,194	**75,522**	86,850	EuroFlow

Spherotech Rainbow Calibration particles (8 peaks) 3.41µm; cat nr. RCP-30-5A, Lot No. EAG01
As per EuroFlow recommendation (31).

SUPPLEMENTARY FIGURES (n=9)

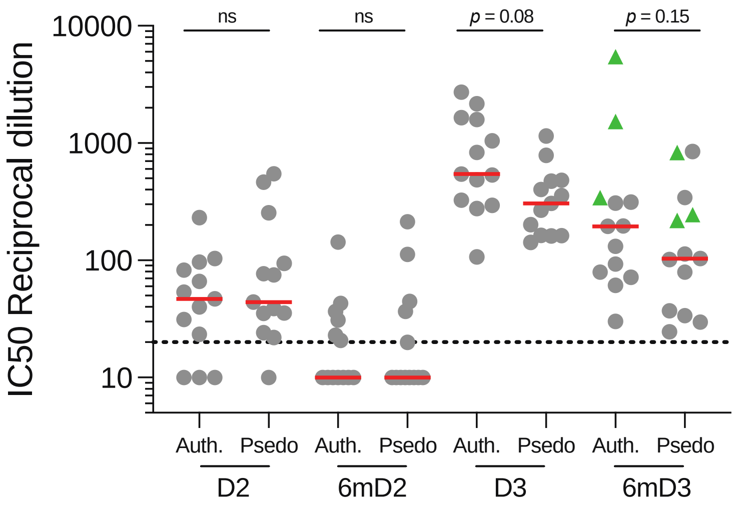

Supplementary Figure 1: Neutralizing antibodies measured by authentic virus or pseudo-neutralization. Neutralizing antibodies from a subset of heterologous vaccinated donors (n = 13) measured using authentic (Auth.) or pseudo virus. Green triangles represent individuals who had a confirmed breakthrough infection (BTI) prior to sampling (**Supplementary Tables 1 and 2**). Red lines represent median values. Wilcoxon matched pairs signed rank test with Bonferroni correction for multiple comparisons.

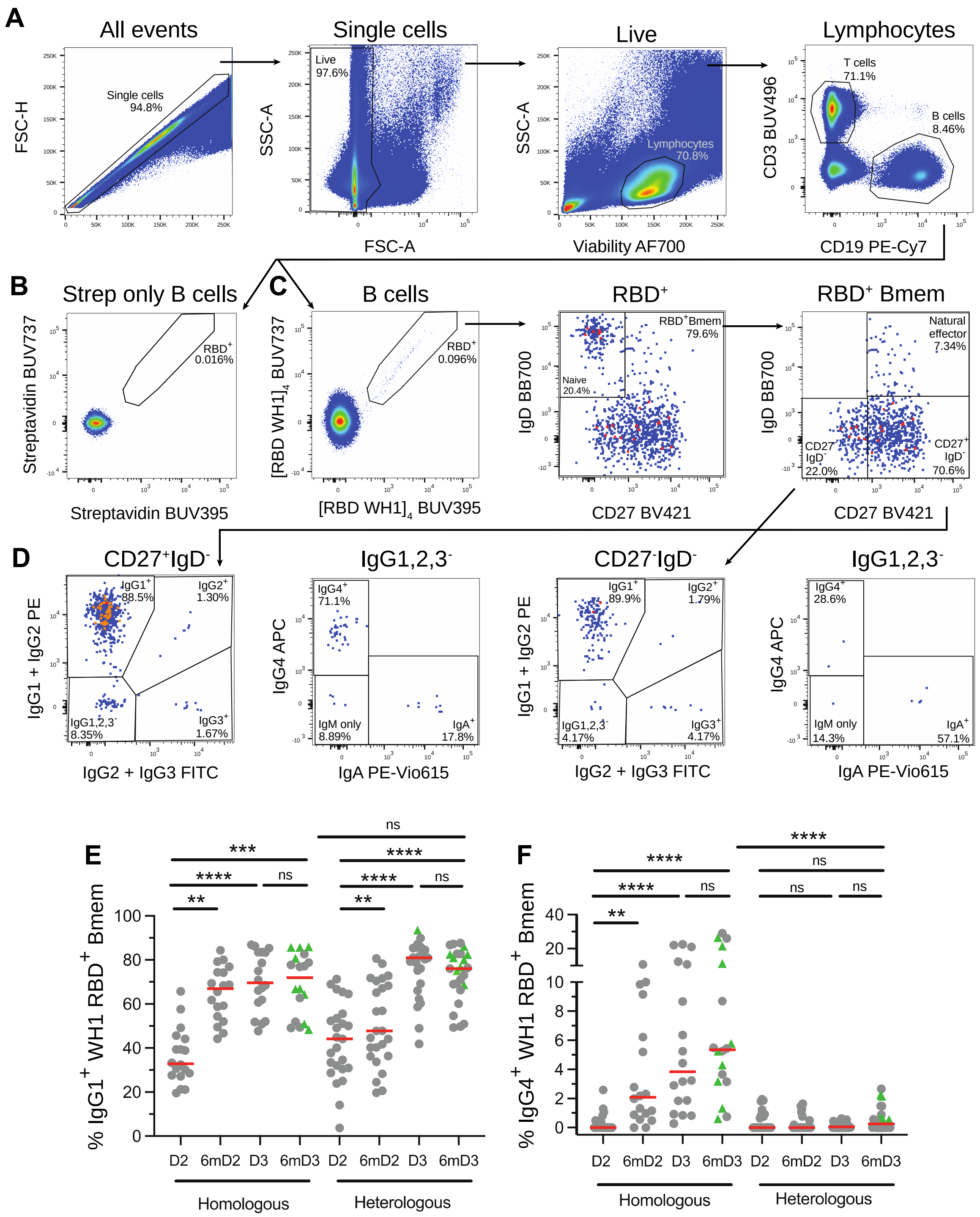

Supplementary Figure 2: Predominant IgG1^{+} ancestral (WH1) RBD-specific Bmem response following homologous and heterologous vaccination. (**A**) Gating strategy to define CD19^{+} B cells. (**B**) B cells in streptavidin only control. (**C**) WH1 RBD-specific B cells, Bmem and CD27^{+} memory. (**D**) CD27$^{+/-}$IgD^{-} RBD-specific Bmem were further separated based on IgG1,2,3,4 and IgA expression. Percentages on each plot are frequencies within the parent gate. Frequencies of (**E**) IgG1^{+} and (**F**) IgG4^{+} RBD-specific Bmem following homologous or

heterologous vaccination. Green triangles represent individuals who had a confirmed breakthrough infection (BTI) prior to sampling (**Supplementary Tables 1 and 2**). Red lines in panels **C** and **D** represent median values. Kruskal-Wallis test with Dunn's multiple comparisons test. ** $p > 0.01$, *** $p > 0.001$, **** $p > 0.0001$.

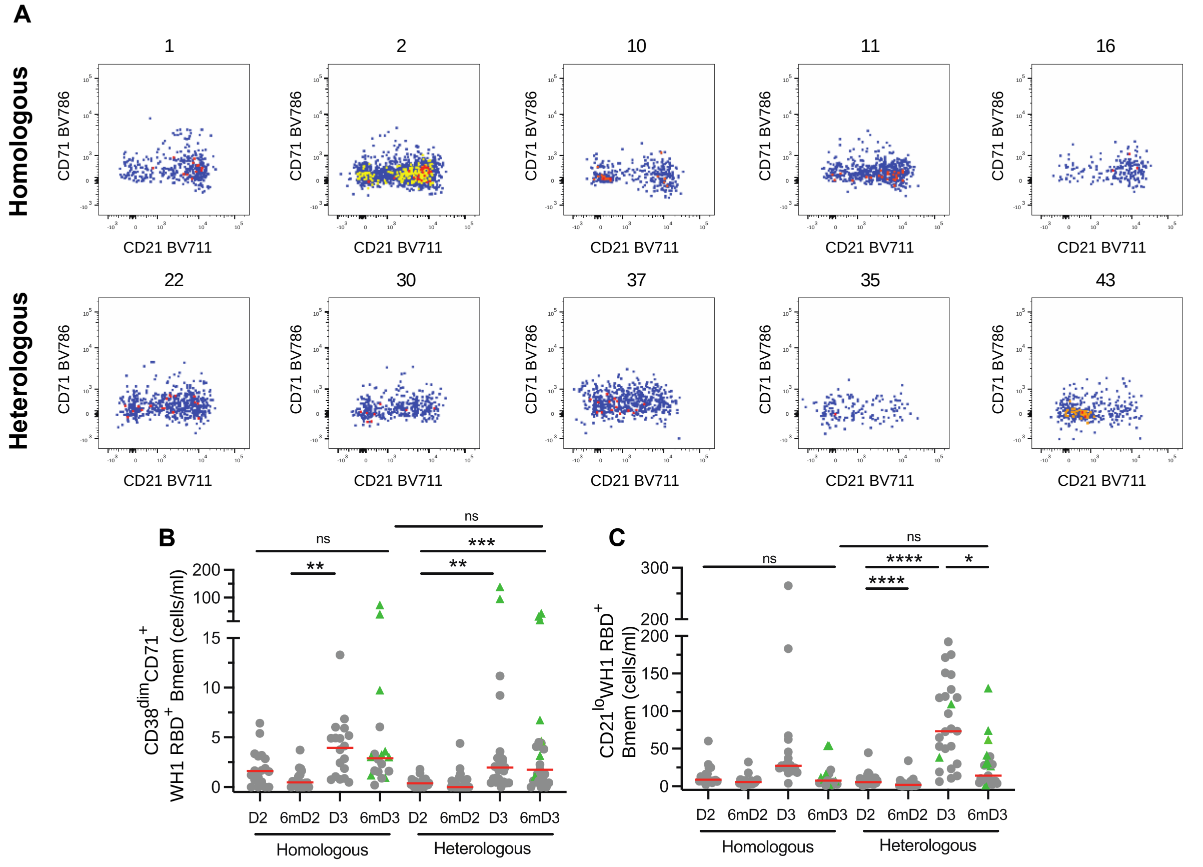

Supplementary Figure 3: Extended phenotype of ancestral (WH1) RBD-specific Bmem following homologous or heterologous vaccination. (A) Expression of CD71 and CD21 on selected donors from the homologous and heterologous cohorts. Numbers above plots represent participant number (**Supplementary Table 1 and 2**). Absolute numbers of (**B**) $CD38^{dim}CD71^{+}$ and (**C**) $CD21^{lo}$ RBD-specific Bmem. Green triangles represent individuals who had a confirmed breakthrough infection (BTI) prior to sampling (**Supplementary Tables 1 and 2**). Red lines represent median values. Kruskal-Wallis test with Dunn's multiple comparisons test. * $p > 0.05$, ** $p > 0.01$, *** $p > 0.001$, **** $p > 0.0001$.

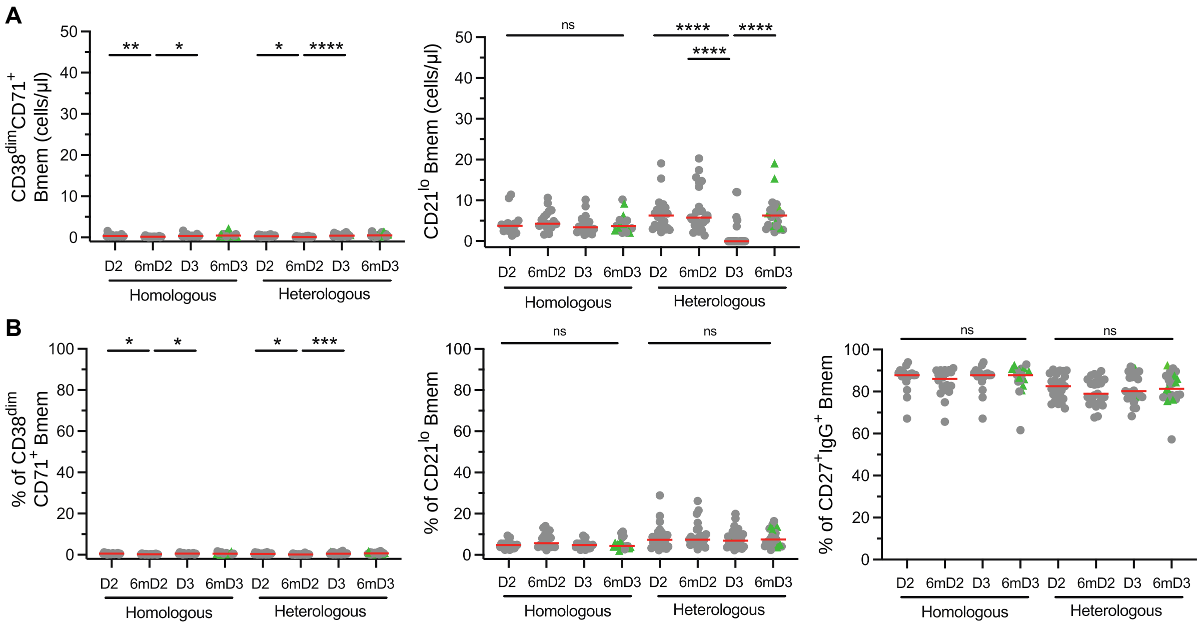

Supplementary Figure 4: Extended phenotype of total Bmem following homologous or heterologous vaccination. (**A**) Absolute numbers of CD38^{dim}CD71^{+} and CD21^{lo} total Bmem Bmem. (**B**) Frequencies of CD38^{dim}CD71^{+}, CD21^{lo} and CD27^{+}IgG^{+} total Bmem. Green triangles represent individuals who had a confirmed breakthrough infection (BTI) prior to sampling (**Supplementary Tables 1 and 2**). Red lines represent median values. Kruskal-Wallis test with Dunn's multiple comparisons test. * p > 0.05, ** p > 0.01, ** p > 0.001, **** p > 0.0001.

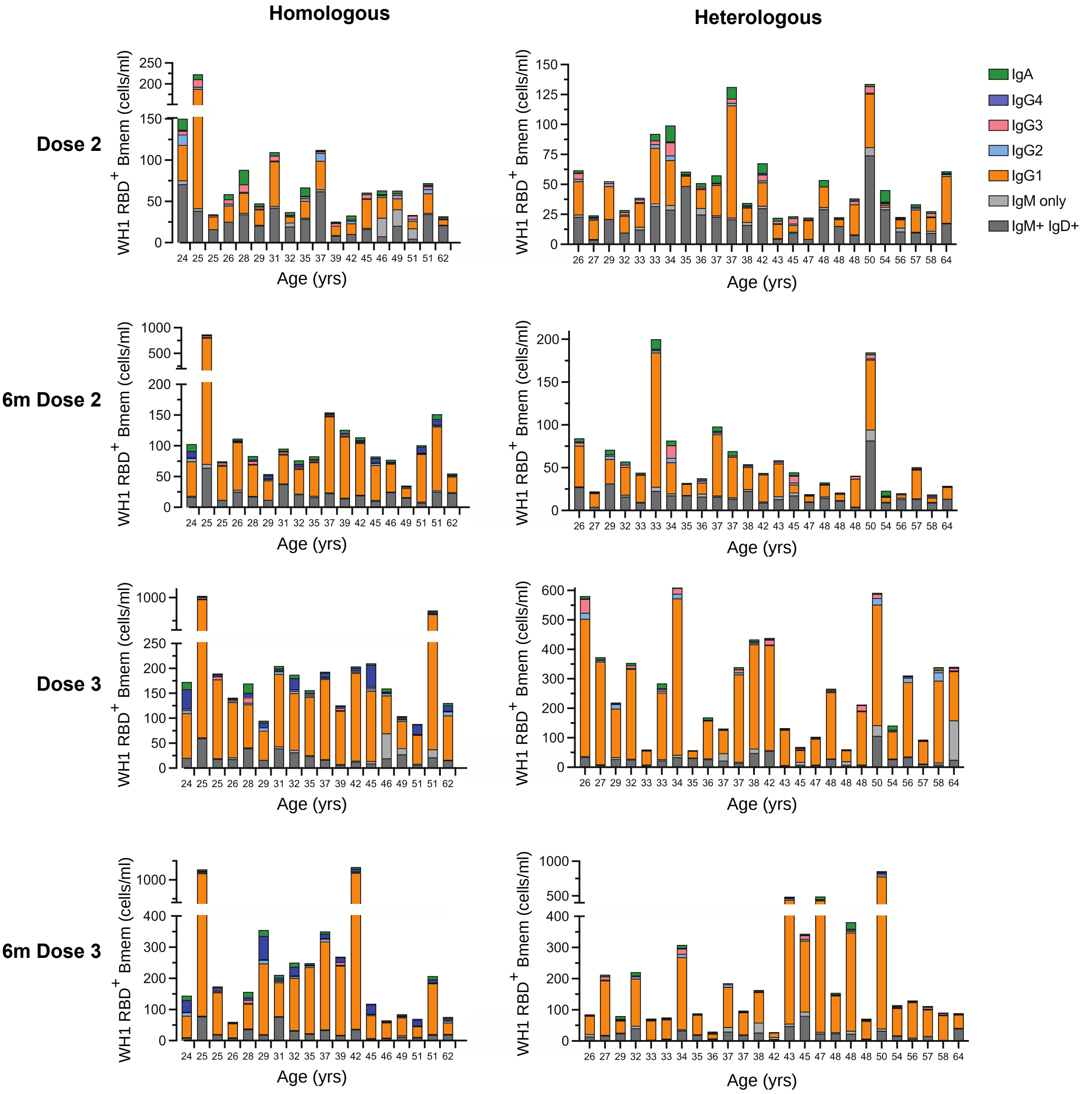

Supplementary Figure 5: Individual ancestral (WH1) RBD-specific Bmem numbers following homologous or heterologous vaccination. Absolute number of RBD-specific Bmem in each individual for the homologous and heterologous vaccination cohorts post-dose 2, 6-months post-dose 2, post-dose 3 and 6-months post-dose 3.

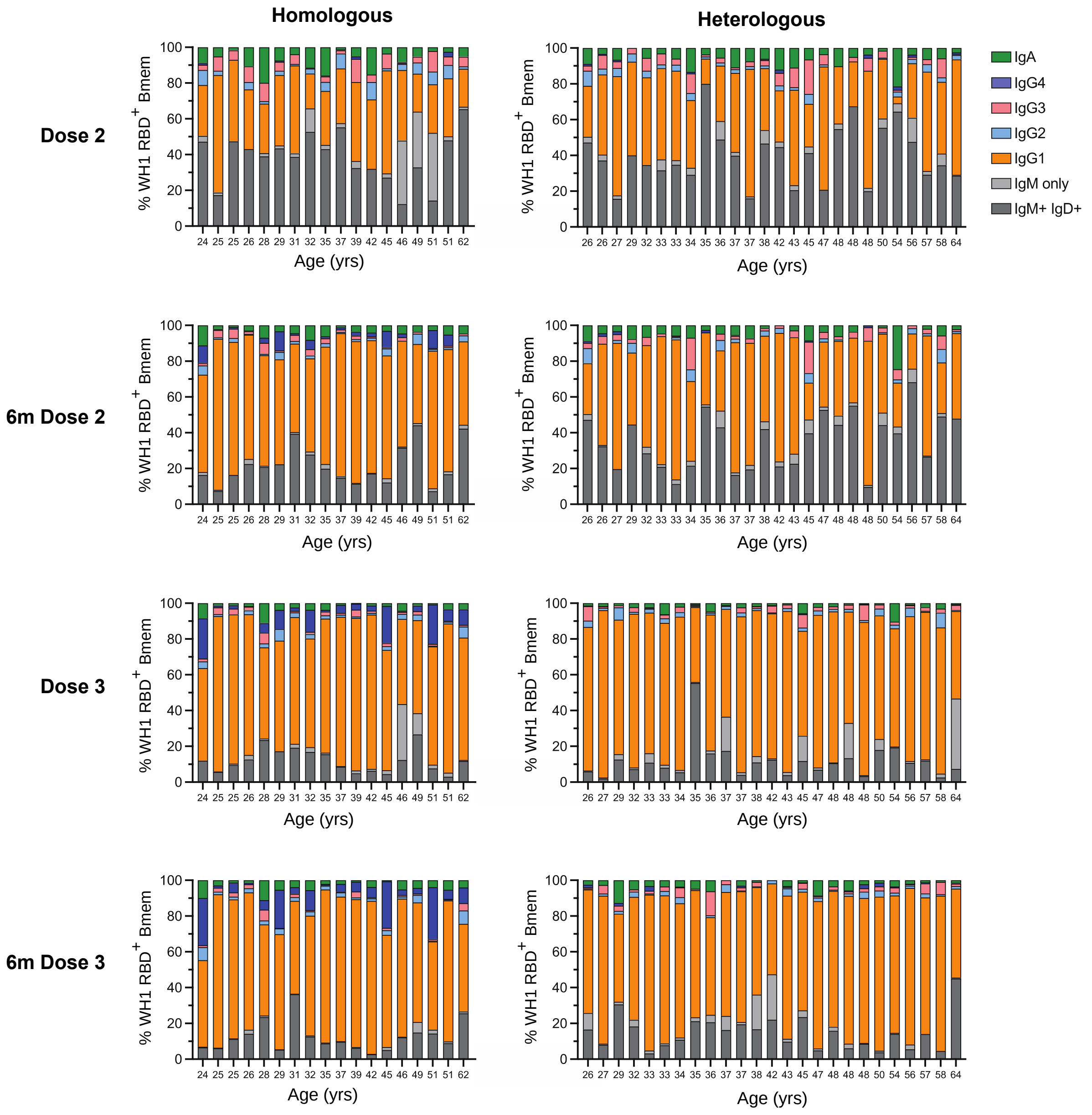

Supplementary Figure 6: Individual frequencies of ancestral (WH1) RBD-specific Bmem following homologous or heterologous vaccination. Frequencies of subsets within WH1 RBD-specific Bmem in each individual for the homologous and heterologous vaccination cohorts 1-month post-dose 2, 6-months post-dose 2, 1-month post-dose 3 and 6-months post-dose 3.

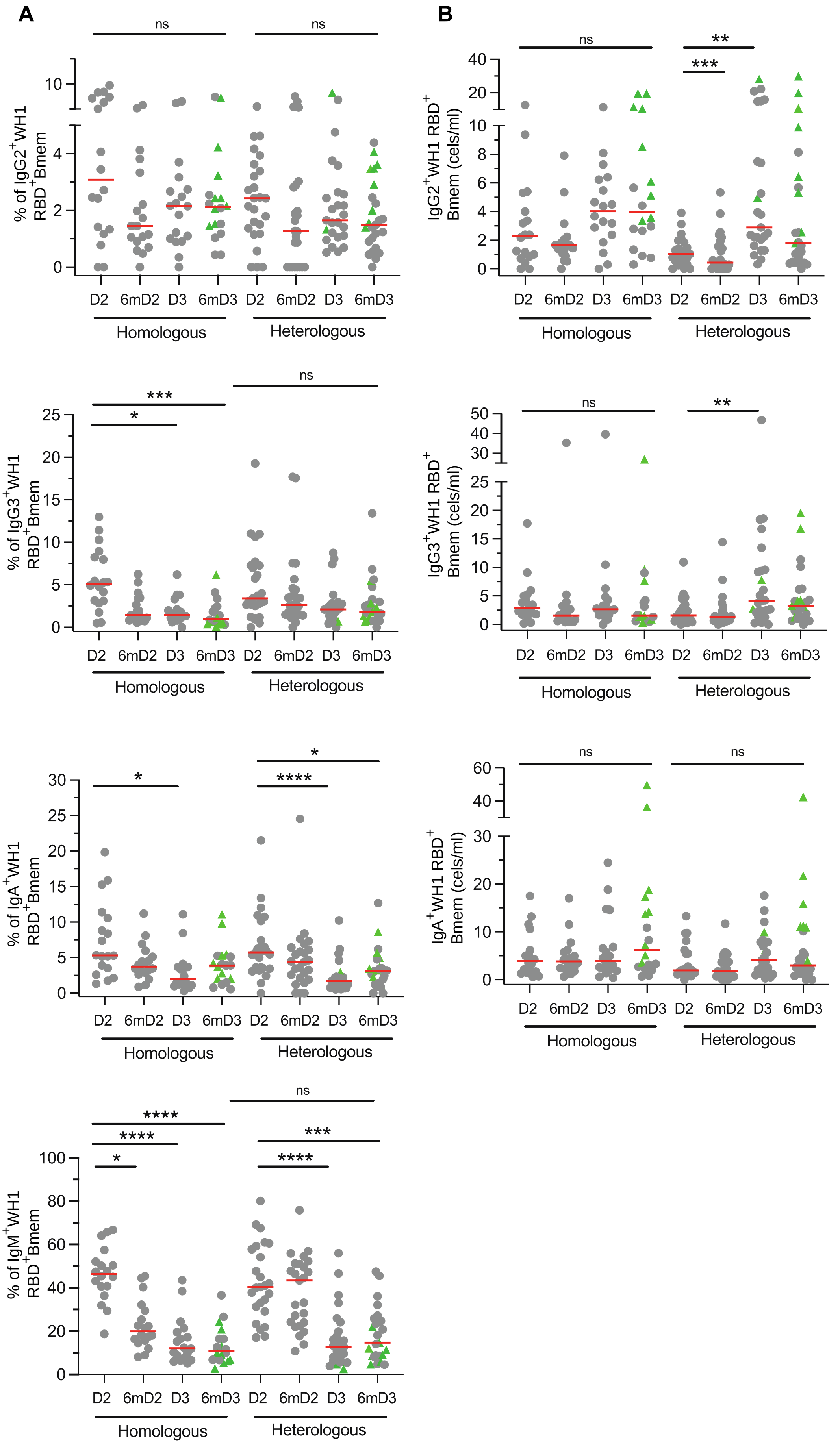

Supplementary Figure 7: Ancestral (WH1) RBD-specific Bmem subsets following homologous or heterologous vaccination. (**A**) Frequencies and (**B**) absolute numbers of

IgG2⁺, IgG3⁺, IgA⁺ and IgM⁺ WH1 RBD-specific Bmem. Green triangles represent individuals who had a confirmed breakthrough infection (BTI) prior to sampling (**Supplementary Tables 1 and 2**). Red lines represent median values. Kruskal-Wallis test with Dunn's multiple comparisons test. * $p > 0.05$, ** $p > 0.01$, ** $p > 0.001$, **** $p > 0.0001$.

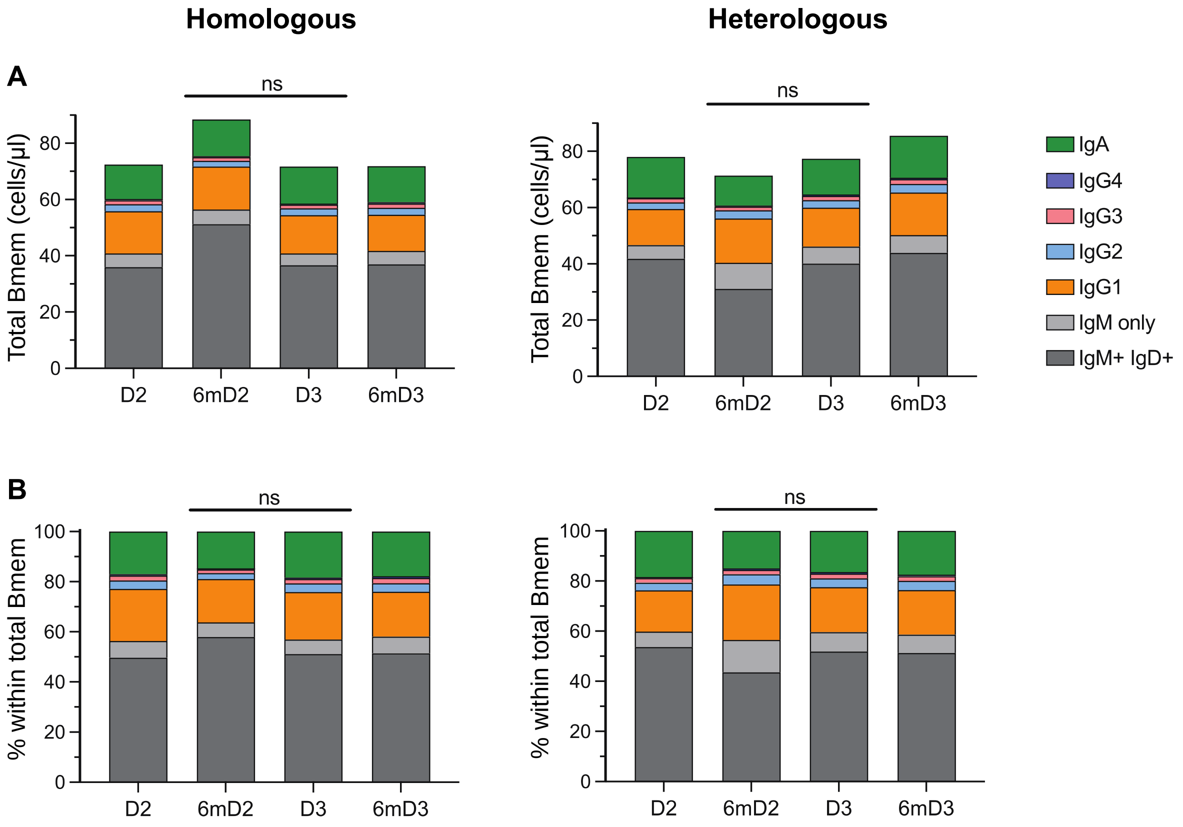

Supplementary Figure 8: Absolute numbers and frequencies of total Bmem Ig isotypes and IgG subclasses following homologous or heterologous vaccination. Median values of (**A**) absolute numbers and (**B**) frequencies of total Bmem following homologous or heterologous vaccination. Kruskal-Wallis test with Dunn's multiple comparisons test.

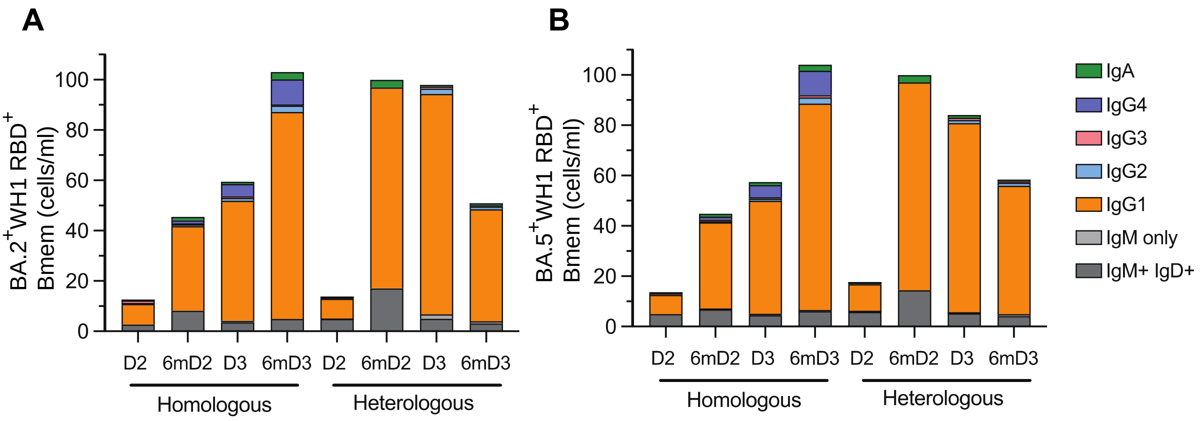

Supplementary Figure 9: Immunophenotype of variant binding RBD-specific Bmem. Median absolute numbers of ancestral (WH1) RBD-specific Bmem that also recognize Omicron (**A**) BA.2 or (**B**) BA.5.
